# Chlorinated pool air, airway mucosal immunity, and respiratory health in competitive swimmers: a mini review

**DOI:** 10.3389/fimmu.2026.1919280

**Published:** 2026-07-28

**Authors:** Jinjing Deng, Xiaochen Li, Liangjing Xia

**Affiliations:** 1 Department of Joint Trauma & Sports Medicine, Sichuan Science City Hospital, Mianyang, Sichuan, China; 2 Department of Surgery, The Third Affiliated Hospital of Shandong First Medical University, Jinan, China

**Keywords:** airway mucosal immunity, chlorinated pool air, competitive swimmers, disinfection by-products, respiratory health, trichloramine

## Abstract

Chlorinated swimming pools are a routine training setting for competitive swimmers, but the breathing zone just above the water surface can contain a variable mixture of volatile disinfection by-products. For athletes who train at high ventilation rates, trichloramine and related compounds may be delivered repeatedly to the airway mucosa, particularly during prolonged sessions with mouth breathing and high humidity. This setting is relevant to immunology because the conducting airway acts as a mucosal barrier as well as a ventilatory conduit, coordinating epithelial integrity, innate immune signaling, allergic responses, and protection against respiratory pathogens. Evidence from swimmer cohorts, pool-worker studies, exposure assessments, and experimental models links chlorinated pool exposure with mucosal irritation, altered lung permeability, airway hyperresponsiveness, neutrophilic or eosinophilic inflammation, and asthma-like symptoms in susceptible individuals. The evidence is nevertheless uneven, partly because studies differ in exposure measurement, training load, atopy, infection status, and biomarker timing. A review focused on this narrow environment may help connect pool chemistry and ventilation with athlete behavior and airway immune outcomes. This mini review shows evidence on chlorinated pool air, airway mucosal immunity, and respiratory health in competitive swimmers, with attention to mechanisms that are clinically relevant for athlete monitoring. This review applies an exposure–barrier–phenotype framework that separates acute peaks from cumulative exposure, ventilation and bather-load effects, and host phenotypes. It also integrates emerging epithelial alarmin and barrier mechanisms, distinguishes disease initiation from exacerbation of asthma or rhinitis, compares pool-derived irritants with common air pollutants, and proposes standardized exposure, biomarker, stratification, and prevention priorities.

## 1 Introduction

Competitive swimming is unusual in sports medicine because much of the training takes place in an enclosed or semi-enclosed pool environment rather than in open air. Indoor pools allow year-round training, temperature control, and disinfection, but chlorination also generates volatile and semi-volatile disinfection by-products when chlorine reacts with sweat, urine, skin cells, cosmetics, and organic nitrogen released by swimmers (1–3). The resulting exposure is often discussed as an irritant or respiratory problem; however, its airway effects are closely linked to mucosal immune function. During intensive swimming, minute ventilation can increase substantially, nasal filtration is partly bypassed through mouth breathing, and swimmers repeatedly inhale from the thin air layer above the water surface, where trichloramine and related compounds may accumulate (4–6). This combination makes competitive swimmers a useful population for examining how a defined training environment interacts with exercise physiology and airway immunity.

Although this exposure setting is small compared with outdoor air pollution, it is clinically important for athletes. Respiratory symptoms, rhinitis, exercise-induced bronchoconstriction, airway hyperresponsiveness, and asthma-like disease are reported more often in swimmers than in some other athletic groups (7–9). Several studies suggest that repeated exposure to chlorination by-products may contribute to airway dysfunction, particularly when high training volume coexists with atopy, poor ventilation, or inadequate pool hygiene (10, 11). The evidence is not fully consistent, and the cardiovascular and metabolic benefits of swimming should be kept in view. The practical question is therefore not whether swimming itself is harmful, but which pool and training conditions increase airway immune risk in susceptible athletes. However, the literature is heterogeneous: occupational studies associate trichloramine with upper and lower respiratory symptoms (6), whereas pediatric and athlete studies differ in whether they detect epithelial leakage, sensitization, or asthma outcomes (33–35, 48, 49). Divergence may reflect exposure misclassification, reverse causation, the healthy-swimmer effect, and failure to distinguish new-onset disease from exacerbation of established asthma or rhinitis.

The airway mucosa is a useful point of entry for this question because it brings together epithelial barrier function, innate immune sensing, local inflammatory recruitment, immunoglobulin responses, and antiviral defense. In athletes, these processes may also be shaped by training load, recovery, sleep, nutritional status, and recent respiratory infection (12–14). Pool exposure research is scattered across occupational hygiene, pediatric allergy, respiratory medicine, and exercise immunology, which makes a focused synthesis necessary. This mini review summarizes evidence on chlorinated pool air as a specific environmental issue in sport and discusses its possible effects on airway mucosal immunity and respiratory health in competitive swimmers. Accordingly, this review explains conflicting results, links pool exposure with contemporary epithelial barrier and alarmin biology, compares pool pollutants with ambient air pollutants, and translates the evidence into standardized research and prevention frameworks.

## 2 Chlorinated pool air exposure during swimming training

Chlorinated pool air should not be treated as a single chemical exposure. It is a changing mixture shaped by water treatment chemistry, swimmer-derived nitrogenous material, pool design, air exchange, humidity, water turbulence, and bather density (15–17). Trichloramine is often emphasized because it is volatile, irritant, and enriched in the air above chlorinated pools, but other disinfection by-products and aerosolized components may also contribute to airway effects. As shown in **Table 1**, a swimmer’s exposure depends on both facility conditions and training behavior, neither of which is captured well by a single pool measurement. A short, intense session in a poorly ventilated indoor pool may therefore produce a different airway challenge from the same training volume in a facility with lower combined chlorine, better air turnover, and reduced bather load.

**Table 1:** Factors affecting chlorinated pool air exposure during competitive swimming training.

Exposure factor	Why it matters for airway immunity
Air-zone trichloramine	A major volatile irritant in indoor chlorinated pools; exposure is most relevant near the breathing zone during high ventilation.
Bather load and hygiene	Swimmer-derived nitrogen increases combined chlorine formation and may raise exposure during crowded training or competitions.
Ventilation and air mixing	Air exchange, flow direction, and removal near the water surface influence what swimmers inhale.
Training intensity and duration	Longer sessions, higher intensity, and repeated daily training increase cumulative airway contact.
Pool design and turbulence	Low ceilings, water agitation, starting blocks, and lane crowding may change conditions close to the breathing zone.
Individual susceptibility	Atopy, asthma history, rhinitis, recent infection, and recovery status may alter the response to a given exposure.

One feature that separates this exposure from many community or occupational exposures is the close coupling of dose with ventilation. Unlike passive pool attendance, competitive training repeatedly increases inspiratory flow, airway cooling and rewarming, and contact between reactive compounds and the epithelial surface (18, 19). Swimmers also breathe close to the air-water interface, where concentrations may differ from measurements taken at ordinary room height. These differences help explain why pool workers, recreational swimmers, children, and elite swimmers should not be treated as interchangeable exposure groups (20, 21). For sports medicine, a useful exposure metric would combine air concentration, breathing pattern, session duration, training intensity, and lane-level conditions rather than relying only on pool-wide water chemistry.

Interpretation is also complicated by temporal variability. Trichloramine and related irritants may rise during crowding, poor shower compliance, competition events, and high water agitation (22). Ventilation systems can reduce average concentrations but still permit localized accumulation near the water surface, especially in indoor facilities with weak air mixing. Repeated measurement is therefore more informative than a single facility audit. For athlete surveillance, environmental monitoring should be paired with symptom diaries, training records, and biomarker timing, because respiratory complaints after high-exposure sessions may otherwise be attributed only to infection, allergy, or nonspecific fatigue. Room-average measurements may miss a stagnant breathing-zone layer; water combined chlorine is operationally useful but is not interchangeable with personal air exposure. Repeated breathing-zone or lane-level measurements should therefore capture peak and time-weighted concentrations alongside session duration, intensity, bather load, and ventilation.

### 2.1 Why current studies produce inconsistent findings

The first source of inconsistency is exposure misclassification. A single pool measurement cannot distinguish an acute peak during a crowded session from a lower but repeated exposure across a season. Studies variously use water chemistry, stationary deck sampling, worker job categories, attendance frequency or self-reported years of swimming as exposure surrogates. These metrics capture different constructs and may attenuate or even reverse apparent dose–response relations. The most informative future metric is an estimated inhaled dose that integrates breathing-zone trichloramine and co-pollutants, peak and time-weighted exposure, session duration, and exercise ventilation (17, 22, 42). The second source is population heterogeneity. Pool workers, recreational swimmers, young children and elite swimmers differ in breathing pattern, cumulative exposure, baseline airway disease and selection into continued participation (20, 21). Within competitive swimming, age, sex, atopy, allergic rhinitis, established asthma, years of training, weekly distance, recent infection and recovery status may modify both mucosal response and symptom reporting. A healthy-worker or healthy-swimmer effect can remove susceptible individuals from the most highly exposed groups, while reverse causation can increase swimming participation among people who select the sport because it is perceived as asthma-friendly. These mechanisms can generate apparently conflicting epidemiological results even when an acute irritant effect is present (48, 49). The third source is outcome and sampling heterogeneity. Symptoms, physician-diagnosed asthma, EIB, bronchial hyperresponsiveness, fractional exhaled nitric oxide (FeNO), sputum cytology, serum club cell protein 16 (CC16) and surfactant proteins do not measure the same biological process. Exercise itself can transiently change airway hydration and inflammatory markers, while delayed sampling may miss a short-lived epithelial response. Allergy season and unrecognized viral infection add further noise. Four controversies remain central: whether typical pool-air exposure can initiate asthma rather than primarily exacerbate susceptible airways; whether repeated transient permeability changes progress to persistent remodeling; whether trichloramine is the dominant causal agent or a marker of a broader mixture; and whether clinically meaningful effects occur below an identifiable exposure threshold.

## 3 Airway epithelial barrier and innate immune responses

**Figure 1:**
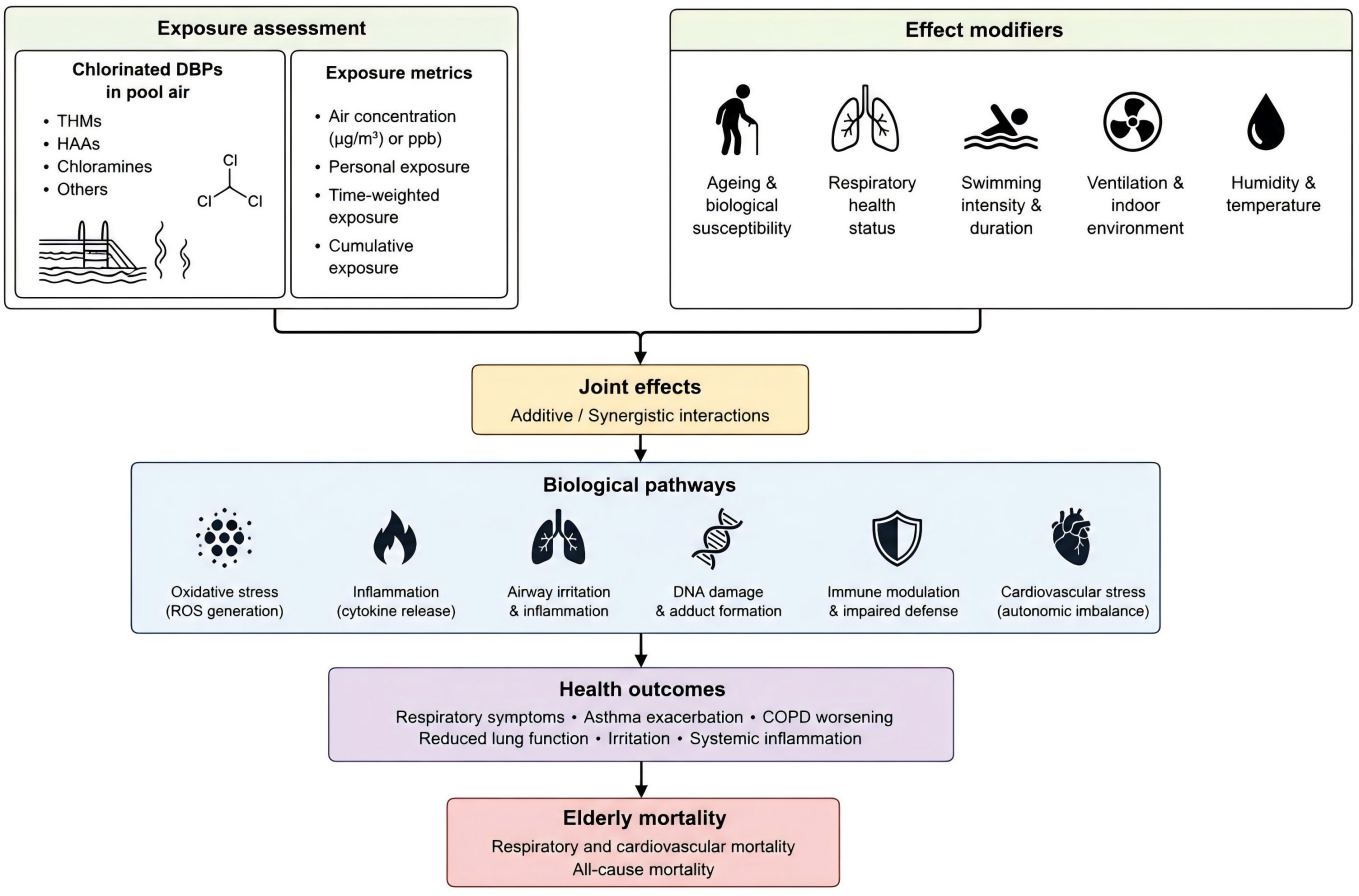
Proposed pathway linking chlorinated pool air exposure with epithelial barrier stress, innate immune activation, and respiratory symptoms in competitive swimmers.

The airway epithelium is the first site of contact for chlorinated pool air. Irritant gases and reactive by-products can disturb epithelial tight junctions, alter mucociliary clearance, increase permeability, and initiate oxidative stress responses (23–25). In swimmer studies, markers of epithelial injury, exhaled nitric oxide, inflammatory cells, and lung permeability have been used to infer airway effects, although findings vary with exposure intensity, athlete age, and measurement timing (26, 27). As shown in **Figure 1**, repeated inhalation of chlorinated pool air can be viewed as a sequence linking chemical irritation and mechanical ventilatory stress with epithelial barrier strain, innate immune signaling, inflammatory cell recruitment, and clinically apparent symptoms. Contemporary barrier research further implicates oxidative modification of junctional proteins, altered airway-surface liquid, and epithelial alarmin signaling (45–47).

### 3.1 Emerging molecular mechanisms and biomarker candidates

Contemporary environmental mucosal immunology suggests several mechanisms that merit direct testing in swimmers. Chlorine, hypochlorous acid and chloramines can inhibit epithelial sodium transport and lung-fluid clearance in experimental systems (46). Oxidant and electrophilic stress may reduce junctional proteins such as zonula occludens-1, occludin and claudins and may trigger epithelial release of interleukin (IL)-33, thymic stromal lymphopoietin (TSLP) and IL-25. These alarmins can activate dendritic cells and group 2 innate lymphoid cells, lower the threshold for T-helper-2/eosinophilic responses, and amplify allergen reactivity (45, 50, 51). Parallel IL-1/IL-8 signaling, neutrophil recruitment and transient-receptor-potential ankyrin 1 sensory activation offer plausible routes to irritant cough, rhinitis and bronchoconstriction (28, 29, 54, 55). Direct evidence for these pathways at typical pool-air doses in competitive swimmers remains limited; high-dose chlorine models should therefore be treated as mechanistic hypotheses, not as quantitative surrogates for routine training exposure.

A useful biomarker strategy should be hierarchical rather than dependent on a single analyte. Exposure should be documented with personal or lane-level trichloramine and, where feasible, a broader volatile disinfection-by-product profile. Barrier injury can be assessed with CC16, surfactant proteins, nasal epithelial integrity and mucociliary measures; inflammatory phenotype with FeNO, induced sputum, nasal lining-fluid IL-33/TSLP/IL-25/IL-8 and differential cell counts; oxidative injury with exhaled 8-isoprostane, hydrogen peroxide or protein chlorination products; and clinical effect with spirometry, bronchodilator response and indirect or direct bronchial challenge. FeNO and exhaled-breath measurements require standardized collection and interpretation (52, 53). No candidate is sufficiently specific for pool exposure in isolation, so concordant change across exposure, barrier, inflammatory and physiological domains is more persuasive than an isolated biomarker shift.

Innate immune responses help explain why irritation, inflammation, and airway hyperresponsiveness often overlap in swimmers. Chlorinated by-products may promote oxidative and electrophilic stress at the epithelial surface, and injured epithelium can release mediators that recruit neutrophils, activate dendritic cells, and amplify local cytokine signaling (28, 29). In athletes, these responses occur in parallel with repeated high-volume ventilation, which may itself induce airway dehydration, osmotic change, and epithelial mechanical stress. Upper respiratory symptoms are therefore not necessarily infectious. Work in endurance athletes has shown that such symptoms may occur without a confirmed pathogen, and swimmer symptoms may reflect inflammatory or irritant mechanisms as well as infection (30, 31). This distinction matters because prevention and treatment differ. When symptoms are mainly viral, pathogen exposure and immune defense are central; when they are driven by irritant-related barrier disruption, pool environment, recovery, and airway protection become more relevant. In practice, both processes may coexist. A swimmer with epithelial irritation may report cough or throat symptoms, while intense training and travel may also influence mucosal host defense and illness susceptibility (32). Few studies have measured epithelial integrity, innate cytokines, viral testing, and precise pool exposure in the same design, which limits causal inference. Even so, the available evidence points to the epithelial barrier as an important link between pool air exposure and athlete respiratory health. Whether repeated irritant episodes remain reversible or establish persistent barrier vulnerability and airway hyperresponsiveness is a central unresolved question.

## 4 Adaptive and allergic immune features in competitive swimmers

Adaptive immunity is relevant because the swimmer respiratory phenotype often includes atopy, rhinitis, bronchial hyperresponsiveness, and asthma-like symptoms. Several epidemiological and clinical reports have linked chlorinated pool attendance with allergic sensitization, airway dysfunction, or asthma outcomes, particularly in susceptible children, elite swimmers, and highly exposed pool personnel (33–35). In competitive athletes, this should not be read as evidence that chlorine exposure alone causes allergic disease. A more cautious interpretation is that repeated epithelial injury may lower the threshold for allergen penetration, antigen presentation, and type 2 immune activation in individuals with pre-existing atopic tendency.

Evidence should be separated by outcome. Worker data link trichloramine exposure with upper- and lower-airway symptoms (5), while pediatric studies report epithelial hyperpermeability, sensitization, or asthma in susceptible groups (33–35, 47). Longitudinal and pooled analyses are weaker or null after accounting for participation and host factors (48, 49). Thus, pool pollutants plausibly exacerbate established asthma, exercise-induced bronchoconstriction, or rhinitis, but evidence for *de novo* asthma at typical training exposures remains context-dependent. Oral early-life or perinatal hypochlorous-acid exposure aggravated allergic airway inflammation in mice through microbiota and arachidonic-acid pathways (44); this model is mechanistically informative but not quantitatively equivalent to inhaled pool air.

The association between pool exposure and allergic immunity is likely to vary with host phenotype. Atopic swimmers may be more sensitive to epithelial disruption, whereas swimmers with established airway inflammation may notice stronger symptoms during high-exposure sessions (36). Serum immunoglobulin patterns, skin-prick testing, fractional exhaled nitric oxide, sputum cytology, and bronchial provocation tests have all been used to describe this phenotype, but they do not measure the same biological process. As shown in **Table 2**, interpretation requires environmental exposure, allergic susceptibility, airway inflammation, lung function, and infection status to be considered separately. Without this separation, swimmers with irritant rhinitis may be grouped with swimmers who have eosinophilic asthma, although their immune mechanisms and preventive needs may differ. Clinically, irritant rhinitis should be distinguished from IgE-associated allergic rhinitis, and objective exercise-induced bronchoconstriction should be separated from eosinophilic asthma; barrier disruption may amplify both nasal and lower-airway allergen responses through IL-33, TSLP, and IL-25 signaling (45, 50, 51).

**Table 2:** Selected immune and respiratory features reported in swimmers exposed to chlorinated pool air, with methodological features relevant to interpretation.

Feature	Examples	Interpretation
Exposure pattern	Single session, repeated weekly exposure, season-long training block	A brief irritant response after one session should be distinguished from sustained barrier stress across a training block.
Respiratory phenotype	Rhinitis, cough, bronchoconstriction, asthma-like disease	Symptoms may arise from irritant, allergic, infectious, or mixed mechanisms.
Epithelial barrier markers	Permeability, epithelial injury proteins, mucociliary symptoms	Barrier disruption is a plausible link between chemical exposure and downstream immune activation.
Inflammatory profile	Neutrophilic, eosinophilic, mixed, or low-grade inflammation	The cell pattern can indicate whether irritant stress, allergic airway disease, or training-related airway strain is more prominent.
Host susceptibility	Atopy, sex, age, training history, prior airway disease	Similar pool conditions may produce different responses across biological and clinical backgrounds.
Training context	Intensity, volume, recovery, travel, competition schedule	Training load can amplify symptoms or alter immune defense, making exposure effects difficult to isolate.
Infection assessment	Viral testing, symptom duration, pathogen confirmation	Infection should be separated from non-infectious airway inflammation when athlete illness is interpreted.
Facility context	Water balance, combined chlorine, ventilation, bather density	These modifiable conditions can guide practical prevention.
Clinical monitoring	Spirometry, bronchial provocation, FeNO, symptom diary	Integrated testing helps avoid both underdiagnosis and overdiagnosis of asthma-like disease.
Prevention strategy	Hygiene, improved ventilation, exposure reduction, medical management	Facility-level changes and athlete-level care are likely to be more useful together than either approach alone.
Exposure ascertainment	Stationary deck sampling, breathing-zone or personal trichloramine, water combined chlorine	Non-equivalent metrics create exposure misclassification and can obscure peak or cumulative dose–response relations.
Sampling timing	Off-exposure baseline; pre-session; immediate, 1–6 h and 24 h post-session	Exercise effects, acute epithelial leakage and delayed inflammation peak at different times; timing must be prespecified.
Comparator and selection	Other athletes, non-athletes, pool workers, within-athlete crossover	Healthy-worker/swimmer effects and reverse causation can bias between-group associations.
Outcome definition	Symptoms, physician-diagnosed asthma, EIB, FeNO, sputum phenotype, CC16/surfactant proteins	These endpoints represent different biological levels and should not be pooled as a single respiratory outcome.

### 4.1 Comparison with common environmental air pollutants

Pool-derived chloramines share with ozone, nitrogen dioxide, diesel exhaust, and particulate matter the capacity to generate oxidative/electrophilic stress, disrupt epithelial junctions and mucociliary function, and amplify alarmin, neutrophilic, dendritic-cell, and innate-lymphoid pathways (45, 50, 51). This common barrier mechanism may explain greater responses in atopic athletes and supports repeated-measures exposure–response designs.

The pool environment is nevertheless distinctive: a humid, rapidly changing volatile mixture is concentrated near the air–water interface, and inhaled dose rises with mouth breathing and exercise ventilation. Particulate pollution has size-dependent deposition and carries heterogeneous constituents, whereas ozone and nitrogen dioxide have different reaction kinetics. Trichloramine may be both irritant and exposure marker; the categories are mechanistically comparable but not interchangeable.

Airway dysfunction is also common in elite sport even when pool exposure is not the main driver. Repeated high-intensity ventilation, cold or dry air exposure in other training settings, and accumulated training stress may contribute to airway inflammation across endurance disciplines (37, 38). What distinguishes swimming is that these exercise-related stresses occur within a chemical environment that can generate reactive irritants. A single-cause explanation is therefore unlikely to be sufficient. A more realistic model is one in which pool air, ventilation, atopy, and training load interact over time, consistent with the broader exercise immunology view that athlete illness and inflammation reflect both external exposures and internal recovery capacity. This interaction model predicts heterogeneous intervention effects: ventilation and precursor control should benefit exposure-linked clusters, while phenotype-guided asthma/rhinitis treatment remains essential when host susceptibility dominates.

## 5 Respiratory symptoms, asthma-like disease, and training implications

Respiratory outcomes in swimmers matter because they influence health, training continuity, race preparation, and medication use. Exercise-induced bronchoconstriction, chronic cough, rhinitis, throat irritation, wheeze, and recurrent upper respiratory symptoms can reduce perceived readiness and lead to repeated interruptions in training (39, 40). Pool-air effects should be considered when symptoms cluster around indoor sessions, worsen during heavy bather load, improve after training breaks, or occur in several athletes using the same facility. These patterns do not prove causality, but they justify including environmental assessment in the sports medicine evaluation. Clinicians should document whether an episode represents new-onset disease, exacerbation of established disease, or a transient irritant response, because the evidentiary and management implications differ.

**Figure 2:**
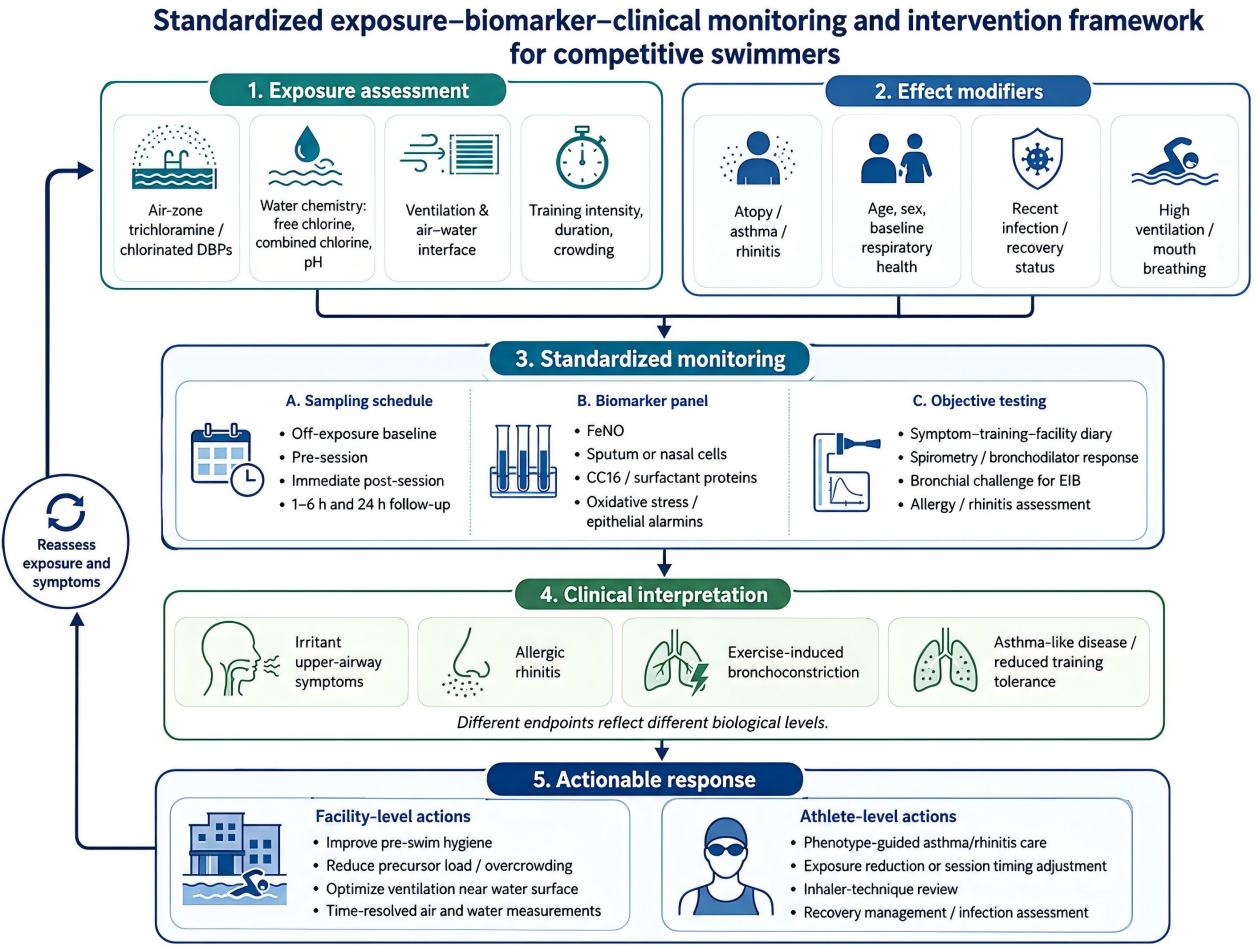
Standardized exposure–biomarker–clinical monitoring and intervention framework for competitive swimmers.

Distinguishing asthma, exercise-induced bronchoconstriction, and nonspecific irritant symptoms is central to athlete care. Objective testing is preferred because symptom reporting alone may underestimate or overestimate airway disease in athletes (41). Spirometry, bronchodilator response, bronchial provocation, FeNO, allergy testing, and selected inflammatory markers can help define phenotype, while environmental data can indicate whether exposure reduction is realistic. As shown in **Figure 2**, a practical monitoring approach would combine pool chemistry, air measurements, ventilation quality, training load, symptoms, lung function, and mucosal biomarkers rather than treating the swimmer only as a patient with isolated asthma-like symptoms. A minimum clinical core should include symptom/exposure timing, spirometry, bronchodilator response, objective bronchial challenge when exercise-induced bronchoconstriction is suspected, and phenotype refinement with FeNO, allergy/rhinitis assessment, and selected nasal or sputum biomarkers (41, 52). Baseline and post-session sampling should be prespecified, as summarized in the revised **Figure 2**.

Prevention should be practical and proportionate. Improved swimmer hygiene before pool entry, reduction of combined chlorine, effective ventilation near the air-water interface, maintenance of water balance, and scheduling adjustments during periods of high bather load are reasonable facility-level strategies (42, 43). At the athlete level, coaches and clinicians should avoid attributing every respiratory episode to poor fitness, weak immunity, or unavoidable seasonal illness. A more useful approach is to look for repeated exposure-symptom patterns and adjust the environment, recovery, and clinical management accordingly. These strategies preserve the benefits of swimming while reducing avoidable airway stress in susceptible athletes. Layered prevention should additionally include pre-swim showering and toilet use, control of overcrowding and precursor load, verification of outdoor-air delivery and near-surface air distribution, and time-resolved air/water measurements when symptoms cluster (42, 43). At athlete level, objective confirmation and phenotype-guided management of asthma, exercise-induced bronchoconstriction, and allergic rhinitis should precede a nonspecific label of “chlorine allergy”; temporary relocation or reduced exposure may be used during exacerbations while the facility is investigated.

## 6 Discussion and future directions

Current evidence suggests that chlorinated pool air is a small but credible environmental concern in sports medicine. Its relevance to immunology lies in the airway mucosa, where epithelial barrier injury, innate immune activation, allergic susceptibility, and respiratory infection defense intersect (23, 28, 32). The main conclusion is not that all chlorinated pools are harmful, but that exposure conditions vary substantially and may become clinically important when high ventilation, repeated training, atopy, and poor air handling occur together. This interpretation is consistent with the heterogeneous respiratory phenotypes reported in swimmers, which cannot be explained by exercise load or chemical exposure alone. The unresolved controversies are initiation versus exacerbation of asthma/rhinitis, reversible permeability versus persistent remodeling, trichloramine as a causal agent versus a marker of a broader mixture, and whether a clinically useful exposure threshold exists.

Several research gaps remain. First, exposure assessment should move beyond occasional water-chemistry measurements toward repeated air-zone measurements at swimmer breathing height. Second, immune biomarkers should be timed in relation to both exposure and exercise, because acute epithelial responses may be missed if sampling occurs too late. Third, infection testing should be incorporated when upper respiratory symptoms are studied; otherwise, irritant symptoms and viral illness will remain difficult to distinguish (30–32). Fourth, future work should stratify by atopy, sex, age, training history, and competitive level, because these factors may modify susceptibility and recovery. These priorities require harmonized exposure, sampling, biomarker, and stratification protocols rather than general recommendations.

A minimum standardized exposure core should include repeated breathing-zone or personal trichloramine measurements that capture peaks and time-weighted averages; concurrent free and combined chlorine, pH, water temperature, bather counts, turbulence, humidity, outdoor-air delivery, and air velocity near the water surface; and session duration, intensity, and estimated minute ventilation. Reporting both air concentration and estimated inhaled dose would distinguish acute high-dose sessions from lower cumulative exposure and allow facility interventions to be evaluated reproducibly.

A standardized biological core should include an off-exposure baseline, a pre-session sample, an immediate or 30–60-minute post-session sample, and a delayed 4–24-hour sample selected for the hypothesized pathway. A practical panel could combine nasal lining fluid, CC16 or surfactant proteins, FeNO, spirometry, induced sputum where feasible, symptom scoring, and viral testing when acute illness is suspected; assay methods, storage, batch correction, and prespecified primary biomarkers should be reported (52, 53). Designs should stratify or test interaction by age, sex, atopy, allergic rhinitis, established asthma or exercise-induced bronchoconstriction, weekly training intensity, cumulative years of swimming, and facility. Repeated-measures crossover studies, season-long cohorts, and pool-level cluster interventions can separate within-athlete exposure effects from stable susceptibility.

The highest-value next step is intervention research. Facility trials should test ventilation and air-distribution changes, precursor-reduction and hygiene programs, bather-load management, and water-treatment modifications using direct breathing-zone exposure as an implementation outcome. Athlete-level trials should evaluate whether phenotype-guided asthma/rhinitis care and temporary exposure modification reduce symptoms without compromising training. Respiratory, biomarker, medication, attendance, and performance outcomes should be reported because a detectable biomarker change is not automatically a clinically meaningful benefit.

Future studies would be stronger if they combined personal or lane-level exposure estimates with clinical respiratory testing, airway inflammatory markers, training load, and symptom trajectories. Such designs could clarify whether pool air acts mainly as an acute irritant trigger, a chronic modifier of airway barrier function, or an amplifier of allergic and infection-related vulnerability. They would also help sports medicine teams determine whether facility-level changes improve athlete health. The practical goal is not to discourage swimming, but to make the training environment safer for athletes who train at high ventilatory loads. Pool-level intervention studies should identify whether environmental changes improve both athlete symptoms and objective airway outcomes.

Chlorinated pool air is a narrowly defined environmental exposure with clear relevance for competitive swimmers. The available literature supports a mechanism in which volatile disinfection by-products, high ventilation, and repeated mucosal contact converge on epithelial barrier stress and airway immune activation. Although causal pathways remain incompletely resolved, a combined exposure perspective can improve athlete monitoring, facility management, and future research. This topic also shows how environmental immunology and sports medicine can meet at a precise mucosal interface, rather than through a broad exposure category. An exposure–barrier–phenotype framework therefore provides a testable bridge between environmental immunology and athlete care.
